# Identification of Long Noncoding RNA Associated ceRNA Networks in Rosacea

**DOI:** 10.1155/2020/9705950

**Published:** 2020-02-24

**Authors:** Lian Wang, Ruifeng Lu, Yujia Wang, Xiaoyun Wang, Dan Hao, Xiang Wen, Yanmei Li, Minghui Zeng, Xian Jiang

**Affiliations:** ^1^Department of Dermatology, West China Hospital, Sichuan University, Chengdu 610041, China; ^2^Department of Pediatrics, West China Second University Hospital, Sichuan University, Chengdu 610041, China; ^3^Department of Pharmacy, Qionglai Medical Center Hospital of Sichuan Province, Chengdu 611500, China

## Abstract

Rosacea is a chronic and relapsing inflammatory cutaneous disorder with highly variable prevalence worldwide that adversely affects the health of patients and their quality of life. However, the molecular characterization of each rosacea subtype is still unclear. Furthermore, little is known about the role of long noncoding RNAs (lncRNAs) in the pathogenesis or regulatory processes of this disorder. In the current study, we established lncRNA-mRNA coexpression networks for three rosacea subtypes (erythematotelangiectatic, papulopustular, and phymatous) and performed their functional enrichment analyses using Gene Onotology, KEGG, GSEA, and WGCNA. Compared to the control group, 13 differentially expressed lncRNAs and 525 differentially expressed mRNAs were identified in the three rosacea subtypes. The differentially expressed genes identified were enriched in four signaling pathways and the GO terms found were associated with leukocyte migration. In addition, we found nine differentially expressed lncRNAs in all three rosacea subtype-related networks, including NEAT1 and HOTAIR, which may play important roles in the pathology of rosacea. Our study provided novel insights into lncRNA-mRNA coexpression networks to discover the molecular mechanisms involved in rosacea development that can be used as future targets of rosacea diagnosis, prevention, and treatment.

## 1. Introduction

Rosacea is a complex chronic and recurrent dermatological condition characterized by flushing, transient/persistent erythema, telangiectasia, inflammatory papules and pustules on the central face, phymatous changes, and ocular manifestations [1, 2]. Rosacea is generally classified into four major subtypes: erythematotelangiectatic (ETR), papulopustular (PPR), phymatous (PhR), and ocular (OR) [2]. Among these subtypes, ETR is the most common one, followed by PPR, PhR, and OR (not discussed in the current work).

Although clinical subtypes are clearly described, the various clinical presentations make the pathophysiology of rosacea elusive; therefore, its precise pathogenesis remains unclear. Several studies suggest that dysregulation of the immune pathways and neurovascular changes are found to varying degrees in different rosacea subtypes [3]. The most studied pathway in rosacea is the cathelicidin activation pathway, followed by inflammasome-associated pathways [3, 4]. T cells response involved in rosacea pathogenesis is dominated by Th1/Th17-polarized immune cells [5].

Long noncoding RNAs (lncRNAs), a class of over 200 nucleotides (nt), are a significant category of ncRNAs involved in a series of biological functions [6]. Abnormal expression of ncRNAs is often related to various human diseases, including cancer, inflammation, and autoimmune diseases. For example, the roles of lncRNAs in cutaneous squamous cell carcinoma [7], atopic dermatitis [8], psoriasis [9, 10], and chronic actinic dermatitis [11] have recently been demonstrated, including immune response, epidermis development, and regulation of leukocyte-mediated cytotoxicity [10, 12]. MicroRNAs (miRNAs), the small ncRNAs with 20–22 nt length, are key controllers of gene expression by targeting messenger RNAs (mRNAs) [13]. Advanced evidence illustrates that lncRNAs and miRNAs not only play important roles in the progression of cutaneous tumor or inflammatory dermatosis separately but also work together to modulate pathways [14–16]. Competing endogenous RNAs (ceRNAs) are noncoding/coding RNAs that compete for the same miRNA to reduce the amount of miRNAs available for interaction via the binding of the miRNA recognition/response elements (MREs); LncRNAs can act as ceRNAs to target and recruit miRNAs [17, 18]. For example, in ovarian cancer, lncRNA (NEAT1) functions as a ceRNA for miRNA506 (miR-506) to promote cell proliferation and migration [19].

To our knowledge, the role of lncRNAs in rosacea, specifically in immune response, is poorly understood. We evaluated the changes in mRNA and lncRNA expression in each rosacea subtype after reannotating microarray data. Our results revealed that both mRNAs and lncRNAs expression profiles varied in each rosacea subtype. Furthermore, we established lncRNA-related ceRNA networks in rosacea to explore the possible correlations among differentially expressed (DE) lncRNAs, miRNAs, and mRNAs. The present study provides a novel and comprehensive overview of the functions and mechanisms of differentially expressed lncRNAs in rosacea pathophysiology.

## 2. Materials and Methods

### 2.1. Microarray Data Process and LncRNA Reannotation

Rosacea gene expression data were obtained from Gene Expression Omnibus (GEO, GSE65914) [5].

There was a total of 58 samples, including 38 rosacea samples (14 ETR, 12 PPR, 12 PhR) and 20 normal samples and each sample consisted of the corresponding RNA-sequence data and mRNA-sequence data. These data were analyzed to identify all gene features in the human genome using a combination of lncRNA reannotation and bioinformatics analysis. Since there were around 1,000 lncRNAs annotated in the library files of Affymetrix HG-U133 plus 2.0 platform [20], we performed the lncRNA reannotation of probes based on the protocols reported previously [21–23]. The retained probes should match the chromosomal position of corresponding lncRNAs retrieved from the GENCODE project (human GENCODE Release 29, GRCh38.p12). The annotated transcripts longer than 200 nt and categorized as “lincRNA,” “antisense,” “processed_transcript,” and “non_coding” were identified as lncRNAs. After removing the multiple annotated probes of both lncRNAs and coding genes, and the lncRNA transcripts with less than three independent probes, we obtained 4,508 lncRNAs for Affymetrix HG‐U133 plus 2.0 microarray. Before processing the differential expression analysis by limma package in R, all the expression values were normalized and log 2 transformed [24].

### 2.2. Putative Target Gene Prediction and Enrichment Analysis

To predict the putative target genes of significantly DE lncRNAs, we applied coexpression and colocation methods. Coexpression was predicted on the basis of the expression correlation coefficient (Pearson's correlation >0.95 or <–0.95). In colocation, all the protein-coding genes 10 kb upstream or downstream of the differently expressed lncRNAs were searched [25]. Gene ontology (GO) enrichment analysis was carried out using the clusterProfiler package [26], and GO terms with corrected *p* values <0.05 were regarded as significantly enriched terms. Kyoto Encyclopedia of Genes and Genomes (KEGG) orthology based annotation system (KOBAS) software was applied to test the significance of DE lncRNAs putative target genes in KEGG pathways (http://www.genome.jp/kegg) [27].

### 2.3. Module Analysis and GSEA

Different modules were identified by a weighted gene coexpression network analysis module (WGCNA) algorithm. A heat map was used to depict the strength of interactions between different modules embedded in R language. All DE genes (DEGs) were included to establish coexpression modules. The “flashclusttool” package was applied to execute the cluster and trait analysis. Clustered Spearman correlation matrix for different RNA-seq replicates for all the groups. Gene set enrichment analysis (GSEA) was used to function annotation of all gene expression data.

### 2.4. Construction of lncRNA-miRNA-mRNA Networks (ceRNA Networks)

The miRcode (http://www.mircode.org/) database was used to predict lncRNA-miRNA interactions, and the miRNAs sequences were modified by StarBase v2.0 database (http://starbase.sysu.edu.cn/) and MicroRNA ENrichment TURned NETwork (MIENTURNET, http://userver.bio.uniroma1.it/apps/mienturnet/) [28]. miRNA targeted mRNAs were obtained from miRDB (http://mirdb.org), miRTarBase (http://miRTarBase.mbc.nctu.edu.tw/), and TargetScan databases (http://www.targetscan.org/vert_71/) [29, 30]. Only mRNAs recognized by all three databases were considered as candidate mRNAs and intersected with the DE mRNAs to screen out the DE mRNAs targeted by the DE miRNAs. Significantly correlated pairs of DE lncRNA-miRNA and miRNA-mRNA interactions were included to create the coexpression network by Cytoscape 3.6.1.

### 2.5. Patients and Specimens

This study was conducted in accordance with the ethical guidelines of the Declaration of Helsinki and approved by the Medical Ethics and Human Research Committee of West China Hospital (No. 2017.163). A total of 7 samples and matched normal adjacent specimens (NAS) used to analyze lncRNAs expression were obtained from patients who were diagnosed with rosacea at West China Hospital, Sichuan University, China. All patients were informed with consent.

### 2.6. Reverse Transcription-Quantitative PCR (RT-qPCR) Assay

Total RNA was extracted from rosacea patients using TRIzol reagent (Invitrogen, USA) and synthesized into cDNA using Reverse Transcription Kits (Applied Biosystems, USA). Quantitative PCR was performed by SYBR PCR kits (Bio-Rad) on Real-Time PCR Detection System (Bio-Rad). RNA concentration was measured by Nanodrop (Invitrogen, USA). Relative lncRNA (HOTAIR, ZNF667-AS1, and NEAT1) levels were normalized against Glyceraldehyde 3-phosphate dehydrogenase (GAPDH) and calculated using the 2^–ΔΔCt^ methods. Each sample was analyzed in triplicate. The primer sequences were as follows:

  GAPDH-F: CGCTGAGTACGTCGTGGAGTC
  GAPDH-R: GCTGATGATCTTGAGGCTGTTGTC
  NEAT1-F: CACAGGCAGGGGAAATGTCT
  NEAT1-R: TGCTGCGTATGCAAGTCTGA
  HOTAIR-F: AGAGAAAAGGCTGAAATGGAGGAC
  HOTAIR-R: AGCTTGGGTGTAATTGCTGGTTT
  ZNF667-AS1-F: TCTCACCTTGTTTTGTCCTGT
  ZNF667-AS1-R: TCGTTTAATGGGTAGAGTTTC

### 2.7. Fluorescence In Situ Hybridization (FISH)

HOTAIR, ZNF667-AS1 and NEAT1 probes were designed and synthesized by RiboBio (Guangzhou, China), and the probe sequences are available upon request

The probe signals were detected with a FISH Kit (RiboBio, Guangzhou, China) according to the manufacturer's instructions. Briefly, rearrangements were analyzed on 5 *μ*m formalin fixed and paraffin embedded (FFPE) sections. Images were captured using a fluorescence microscope (Leica, Germany). At least 50 nuclei were scored for each probe and case.

### 2.8. Statistical Analysis

All statistical analyses were conducted on Graph Prism 7. The data measurements were presented as mean ± standard deviation (SD). Statistical analyses were carried out by Student's *t*-test and one-way ANOVA as appropriate. *p* < 0.05 was considered as significant.

## 3. Results

### 3.1. Reannotation of lncRNAs and Bioinformatics Analysis of DE RNAs

Expression levels of lncRNAs and mRNAs were used to produce heat maps

**Figure 1:**
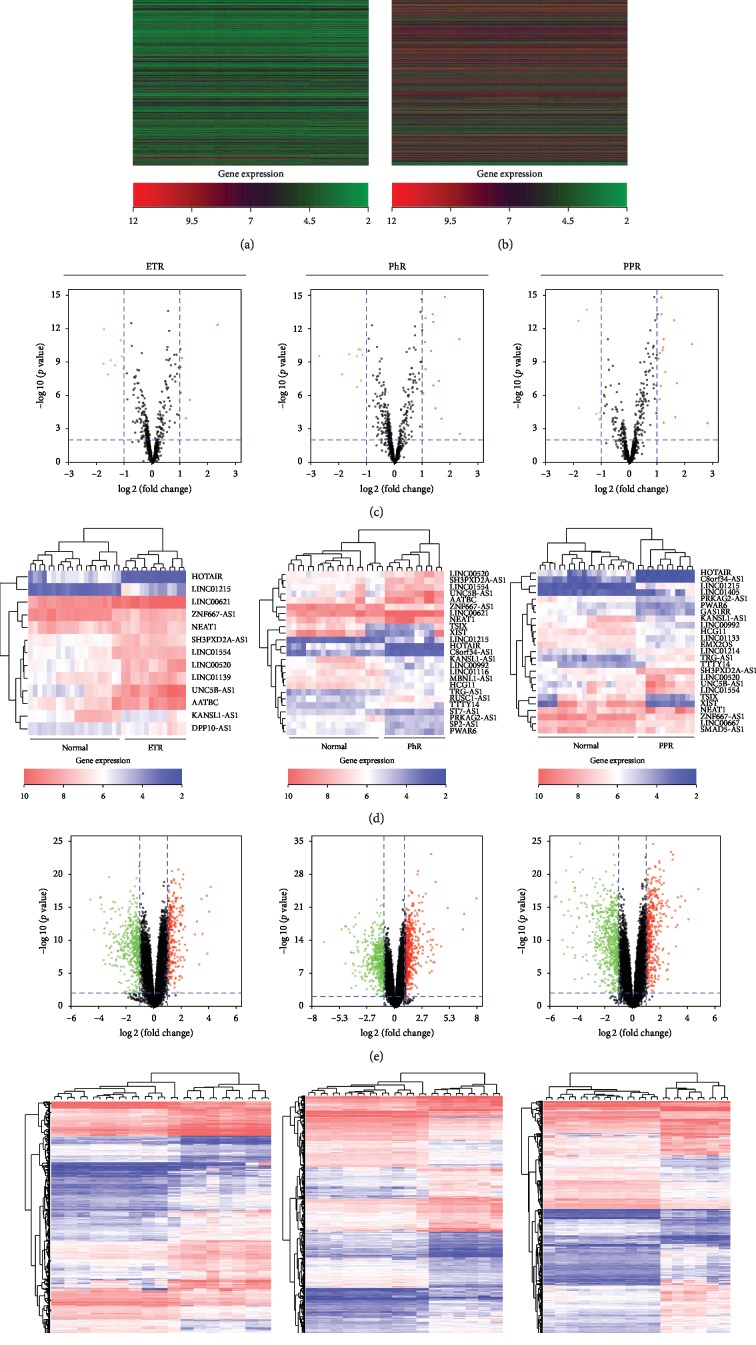
LncRNA and mRNA expression profile of normal group, ETR, PhR, and PPR groups. (a) Heat map of lncRNAs expression in normal group, ETR, PhR, and PPR groups. Red represents upregulated lncRNAs and green represents downregulated lncRNAs. (b) Heat map of mRNAs expression in normal group, ETR, PhR, and PPR groups. Red represents upregulated mRNAs and green represents downregulated mRNAs. (c) Volcano plots of lncRNAs for normal group, ETR, PhR, and PPR groups. The horizontal axis represents fold change (log 2) and the vertical axis is *p* value (–log 10). Red points (fold change > 1) indicate upregulated lncRNAs and blue points (fold change < –1) indicate downregulated lncRNAs. (d) Hierarchical clustered heat maps showing the log 10 transformed expression values for differently expressed lncRNAs among normal group, ETR, PhR, and PPR groups. The horizontal axis represents samples, while the vertical axis represents the biological elements studied. Red represents higher expression and blue represents lower expression. (e) Volcano plots of mRNAs for normal group, ETR, PhR, and PPR groups. The horizontal axis is fold change (log 2) and the vertical axis is *p* value (–log 10). Red points (fold change > 1) indicate upregulated mRNAs and blue points (fold change < –1) indicate downregulated mRNAs. (f) Hierarchical clustered heat maps showing the log10 transformed expression values for differently expressed mRNAs among normal group, ETR, PhR, and PPR groups. Red represents higher expression and blue represents lower expression.

Both lncRNAs and mRNAs were self-segregated into three subtypes of rosacea and normal groups, as shown in Figures 1(a) and 1(b). Volcano plots estimated the statistical significance of differentially expressed (DE) lncRNAs and mRNAs (Figures 1(c) and 1(e)). Subsequently, DE lncRNAs and mRNAs were screened between different subtypes of rosacea and normal groups by unsupervised hierarchical clustering analysis (Figures 1(d) and 1(f)). We identified 13 DE lncRNAs between the normal and ETR groups that were statistically significant. Five out of 13 were upregulated and eight downregulated. Moreover, there were 25 statistically significant DE lncRNAs between the normal and PhR groups, of which 15 were upregulated and 10 downregulated. In addition, the number of significant DE lncRNAs between the normal and PPR groups was 25, where 18 lncRNAs were upregulated and 7 downregulated. Regarding mRNAs, we identified 777 DE mRNAs with statistical significance between the normal and ETR groups, of which 505 genes were upregulated and 272 downregulated. Furthermore, a total of 1051 DE mRNAs showed statistical significance between the normal and PhR groups, with 532 upregulated genes and 494 downregulated genes. Finally, there were 1069 DE mRNAs with statistical significance between the normal and PPR groups, among which 643 genes were upregulated and 426 were downregulated.

**Figure 2:**
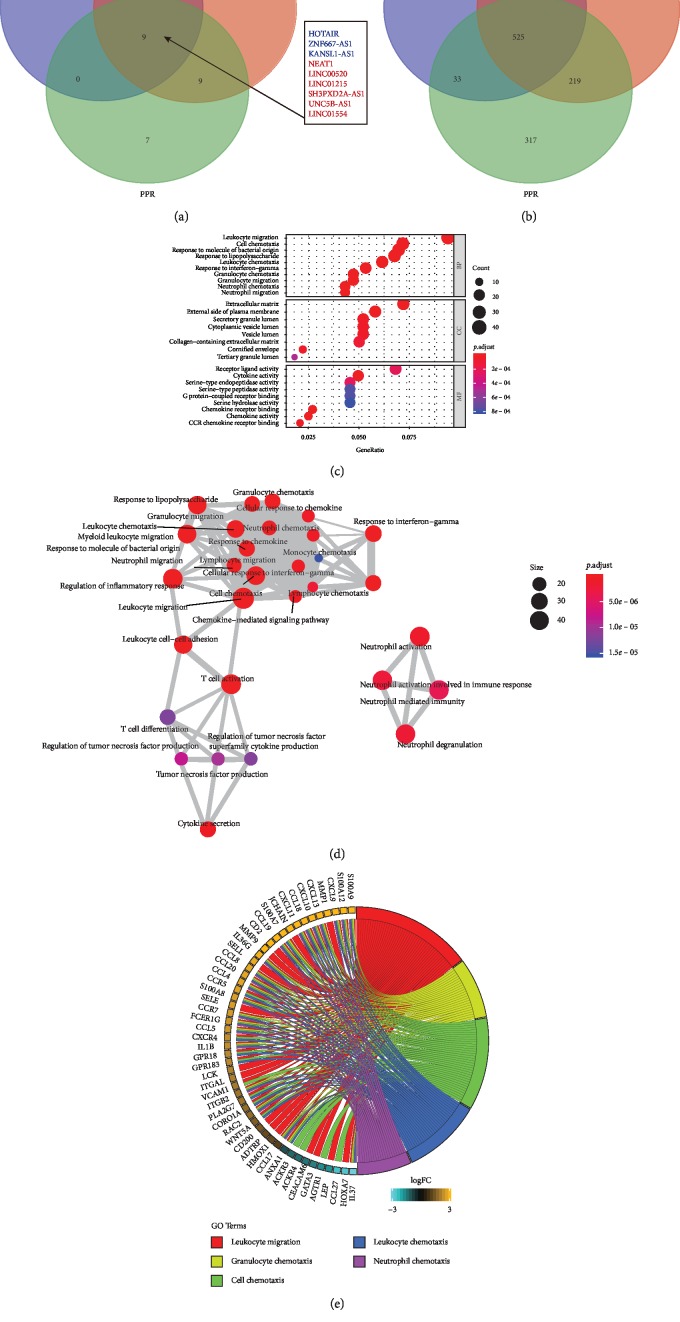
(a) The overlapping Venn diagram of lncRNAs among ETR, PhR, and PPR groups. There are 6 upregulated lncRNAs and 3 kinds of downregulated lncRNAs. (b) The overlapping Venn diagram of mRNAs among ETR, PhR, and PPR groups. (c) Gene ontology (GO) analysis of differentially expressed lncRNAs in rosacea patients and normal group. The horizontal axis represents the proportion of those genes accounted for in all the GO annotated genes, the left side of the vertical axis represents the annotation terms, and the right side of the vertical axis represents biological process (BP) terms, cellular component (CC) terms, and molecular function (MF) terms. Bubble scale represents the number of genes in each GO term; depth of bubble color represents *p* value. (d) The annotation terms are displayed as an interaction network using the BinGO plug-in for Cytoscape. Bubble scale represents number of genes; depth of bubble color represents *p* value. (e) The enriched GO biological process terms of differentially expressed mRNAs involved in the lncRNAs network.

The overlapping Venn diagram showed that six DE lncRNAs identified were significantly upregulated, while three were significantly downregulated in all rosacea subtypes (Figure 2(a)). Besides, 525 differentially annotated mRNAs were obtained in all subtypes of rosacea (Figure 2(b)). The functions of differentially expressed lncRNAs were predicted through the GO analysis. Trans targets were enriched in ten biological processes (BP), in eight cellular components (CC), and in nine molecular functions (MF) (Figure 2(c)). GO analysis in different groups are shown in Figures [Supplementary-material supplementary-material-1], [Supplementary-material supplementary-material-1], and [Supplementary-material supplementary-material-1]. Most significant enriched BP identified in ETR group were leukocyte migration, epidermis development, and skin development. The top three enriched BP identified in PhR group were T cell activation, leukocyte migration, and neutrophil activation. Finally, leukocyte migration, cell chemotaxis, and leukocyte chemotaxis were the most significant enriched BP identified in PPR. All enriched GO terms were displayed as an interaction network (Figure 2(d)), which were divided into three categories: leukocyte migration, neutrophil activation, and T cell activation.

Enriched GO biological processes of DE mRNAs involved in the lncRNAs network are shown in Figure 2(e). Forty and ten genes were up- and downregulated, respectively. Most of these genes are associated with chemotaxis and migration. Different candidate genes identified in the three most significant BP coexpression network in the three rosacea subtypes were represented in Figures [Supplementary-material supplementary-material-1], [Supplementary-material supplementary-material-1], and [Supplementary-material supplementary-material-1].

### 3.2. Construction of Cluster Dendrogram and WGCNA

**Figure 3:**
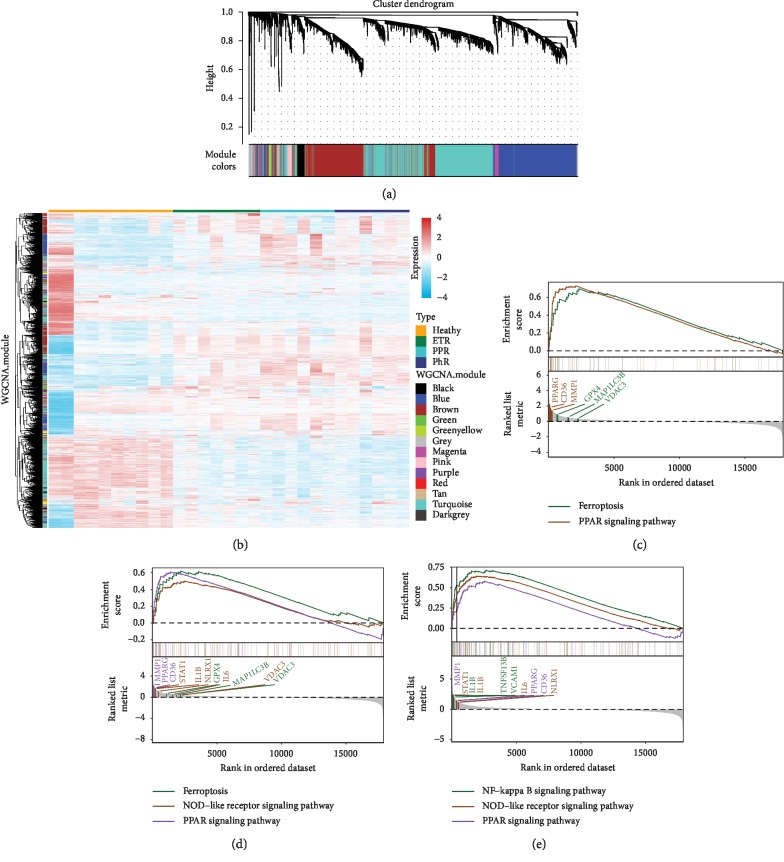
(a) Clustering dendrogram of genes. The dissimilarity of genes is based on the topological overlap. The genes are assigned to different modules and are identified using different colors. (b) Interactions between genes in the coexpression modules. The different colors on the horizontal and vertical axis represent different groups and modules. The colors in the middle represent the relativity among each module. (c) Gene set enrichment analysis (GSEA) in differentially expressed mRNAs linked to lncRNAs in the ETR group. Ferroptosis regulated by GPX4, MAP1LC3B, VDAC3 and PPAR signaling pathway regulated by PPARG, CD36, MMP1 were involved. The horizontal axis represents the rank in all the ordered dataset, and the vertical axes represent enrichment score and ranked list metric. (d) Ferroptosis regulated by GPX4, MAP1LC3B, VDAC3, NOD-like receptor signaling pathway regulated by STAT1, IL1B, NLRX1, IL6, VDAC3, and PPAR signaling pathway regulated by PPARG, CD36, MMP1 were involved in PhR group. (e) NF-kappa B signaling pathway regulated by IL1B, TNFSF13B, VCAM1, NOD-like receptor signaling pathway regulated by STAT1, IL1B, IL6, NLRX1, and PPAR signaling pathway regulated by MMP1, PPARG, CD36 were involved in PPR group.

**Figure 4:**
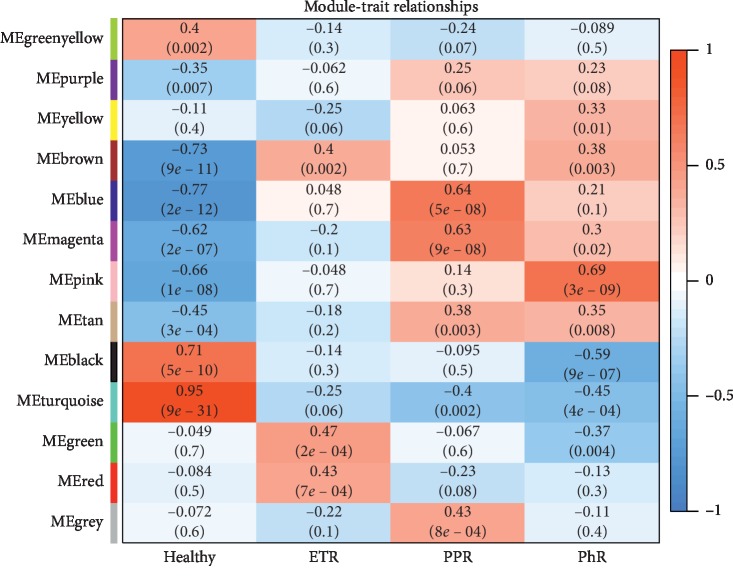
Associations of consensus module eigengenes and the clinical phenotype of rosacea. The module name is displayed in the left-hand panel. Numbers in the table indicate the correlations of the corresponding module eigengenes and clinical phenotype, with *p* values displayed below the correlations in brackets. The intensity and direction of associations are indicated on the right side of the heatmap (red, positively correlated; blue, negatively correlated). ME, module eigengene.

We identified thirteen distinct coexpressing gene modules in rosacea groups. Next, we constructed modules with different colors and clustered the DEGs (Figure 3(a)). Interactions between genes by weighted gene coexpression network analysis (WGCNA) module illustrated that there were notable differences in the correlation between normal and the three rosacea subtypes, especially in the PPR group (Figure 3(b)). The association between clinical phenotypes and genome-wide expression profiles was studied using “Eigengene Networks” tool (Figure 4). Here, the “green” and “red” modules were highly associated with ETR. On the other hand, the “blue,” “magenta” and “grey” modules were highly associated with PPR and the “pink” module was most highly associated with PhR.

Gene set enrichment analysis (GSEA) was conducted on the genes in the thirteen constructed modules (Figures 3(c)–3(e)). Genes in ETR group were mainly enriched in ferroptosis and PPAR signaling pathway. The core regulators on ferroptosis included GPX4, MAP1LC3B, and VDAC3 genes. On the other hand, the core regulators on PPAR signaling pathway were PPARG, CD36, and MMP1 genes. Similarly, genes in PhR group were mainly enriched in ferroptosis, NOD-like receptor, and PPAR signaling pathways. The core regulators genes on NOD-like receptor signaling pathway were STAT1, IL1B, NLRX1, IL6, and VDAC. Genes in PPR group were enriched in NF-κB, NOD-like, and PPAR signaling pathways. IL1B, TNFSF13B, and VCAM1 genes were regulators of NF-κB signaling pathway.

### 3.3. Construction of the ceRNA Network and GO Analyses and KEGG Pathway

**Figure 5:**
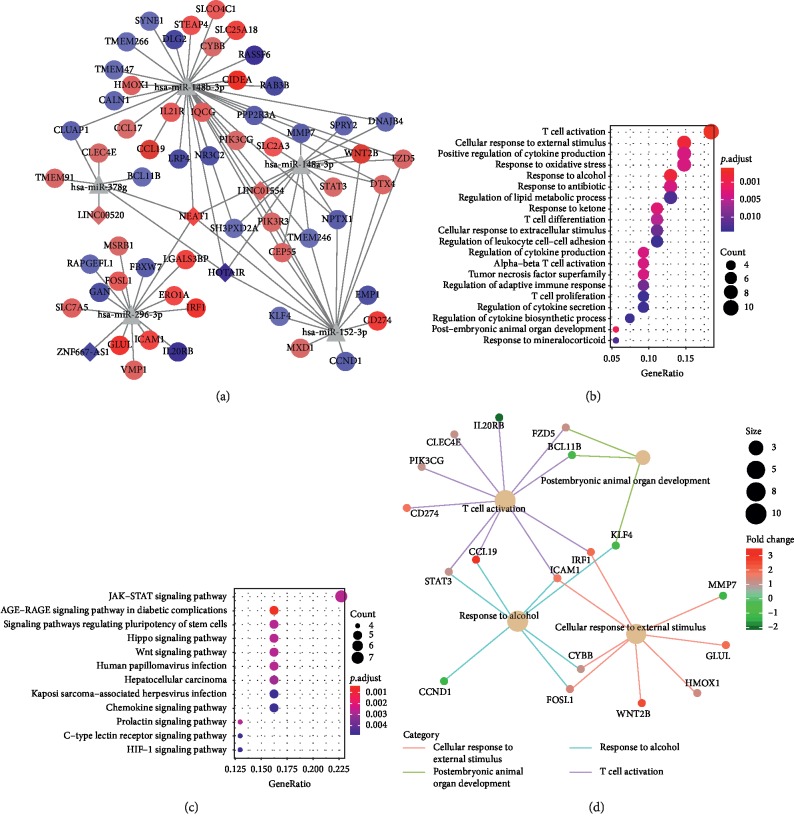
(a) Global view of the ceRNA network in the turquoise module. The circle depicts mRNA, triangle depicts miRNA, and diamond depicts lncRNA. Red and blue depict up- and downregulated genes, respectively. (b) The GO analyses. (c) The KEGG pathway enrichment. (d) GO terms that were associated in the ceRNA network are displayed as an interaction network. Lines indicate GO terms. Bubble scale represents the number of genes. The depth of bubble color represents log 2 fold change.

LncRNA-miRNA interactions and miRNA-mRNA interactions were combined to establish a complete lncRNA-miRNA-mRNA network, which consisted of 5 lncRNAs, 5 miRNAs, and 56 mRNAs, totaling to 241 interactions (Figure 5(a)). LncRNAs (NEAT1, HOTAIR, and ZNF667-AS1) acted as ceRNAs to target miRNAs (hsa-miR-148b-3p, hsa-miR-148a-3p, hsa-miR-296-3p, hsa-miR-378g, and hsa-miR-152-3p), which play important roles in this network. Upregulation of mRNAs (e.g., CCL19, IL21R, WNT2B, STAT3, ICAM1, GLUL) and downregulation of mRNAs (e.g., IL20RB, KLF4, SH3PXD2A) had strong connections with these miRNAs in the network. The GO analyses revealed T cell activation, cell response to an external stimulus, and response to alcohol (Figure 5(b)). The KEGG pathway revealed JAK-STAT signaling pathway (Figure 5(c)). The interaction network on GO terms illustrated major GO terms were interacted with various mRNAs (Figure 5(d)).

### 3.4. RT-qPCR and FISH

**Figure 6:**
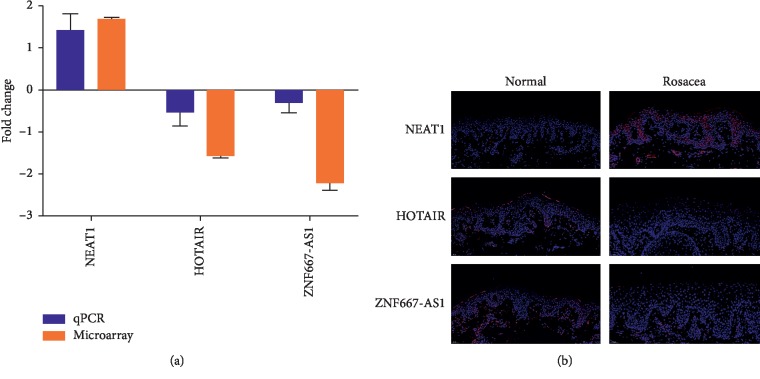
(a) The relative expression of NEAT1, HOTAIR, and ZNF667-AS1 in rosacea lesions by qPCR and microarray. (b) FISH staining (Merge) confirmed the expression of NEAT1 and decreased expression of HOTAIR and ZNF667-AS1 in rosacea compared to normal adjacent specimens.

The results by RT-qPCR revealed the increased expression of NEAT1 and decreased expression of HOTAIR, ZNF667-AS1 in rosacea tissues, which was consistent with microarray (Figure 6(a)). FISH staining (Merge) confirmed the increased expression of NEAT1 and decreased expression of HOTAIR and ZNF667-AS1 in rosacea compared to normal adjacent specimens (Figure 6(b)).

## 4. Discussion

The exact molecular mechanisms of rosacea are unclear; however, this disorder is expected to be the result of a combination of several causes with genetic predisposition [3]. Accumulating evidence points to activated cellular pattern recognition receptors, such as toll-like receptor 2 (TLR2), transient receptor potential ion channels, and inflammatory mediators as actors of critical steps in the clinical diagnosis of rosacea [31]. The cathelicidin activation pathway, initiated by activated TLR2, leads to upregulation and release of matrix metalloproteases (MMPs) and kallikrein 5 (KLK5) [32]. MMPs and KLK5 participate in a proteolytic cascade and activate the cathelicidin peptide LL-37 from its inactive precursor, inducing a sequence of proinflammatory events, such as leucocyte chemotaxis, activation of NF-κB, and angiogenesis [33]. Besides, the inflammasome components NOD-like receptor 3 and caspase-1 can activate interleukin-1*β* (IL-1*β*). Both NF-κB responsive and inducible genes enhance the expression of tumor necrosis factor (TNF), IL-8, and cyclooxygenase-2, which mediate neutrophil chemotaxis, inflammation amplification, prostaglandin E2 synthesis, and downstream vascular effects [34, 35].

Dysregulation of lncRNAs has been reported to be associated with several human diseases, including autoimmune diseases and different types of tumors [7, 8, 21, 36–39]. Recently, lncRNAs broad expression in cutaneous disorders including systemic lupus erythematosus (SLE), sensitive skin, psoriasis, and aging process has been documented [40–43]. These results raise the possibility that lncRNAs play central functional roles in the pathology of inflammatory and immune disorders. Moreover, these effects would be mainly mediated by the NF‐κB, AP‐1, focal adhesion, and PI3K-Akt signaling pathways [41, 42]. However, the relationships between the lncRNA-mRNA network and rosacea have not been elucidated so far. We applied an integrated bioinformatics approach to establish the ceRNA network, RT-qPCR, and FISH to identify dysregulated lncRNAs that may play important roles in rosacea from the database.

miRNAs function in RNA silencing and posttranscriptional regulation of gene expression. Recent advances reveal miRNAs are associated with inflammatory skin disease (e.g., psoriasis) [15]. In the current study, a total of 5 DE-miRNAs were screened out, including hsa-miR-148b-3p, hsa-miR-148a-3p, and hsa-miR-296-3p, which targeted the most genes. Furthermore, the three miRNAs were significantly enriched in T cell activation and JAK-STAT signaling pathway. Besides, in the present study, we observed an enrichment of lncRNAs in leukocyte migration and cell chemotaxis in the three rosacea subtypes. This indicates an increase in innate immunity and inflammatory reactions in rosacea pathophysiology. The interaction network obtained suggests that T cell activation plays an important role in rosacea development. Similarly, enrichment analyses showed that genes mainly involved in chemotaxis and migration of cells were upregulated (including S100A9, S100A12, CXCL9, MMP1, and CXCL13) and downregulated (including IL37, HOXA7, CCL21, LEP, and AGTR1). These data indicate the importance of these processes in the pathogenesis of rosacea.

In addition, KEGG pathway enrichment analyses reveal that ferroptosis, PPAR, NOD-like, and NF-κB and JAK-STAT signaling pathways are involved in rosacea. Ferroptosis is a distinct form of programmed cell death that is associated with the generation of iron-dependent reactive oxygen species (ROS) [44]. Peroxisome proliferator‐activated receptors (PPARs) are ligand-activated transcription factors, expressed primarily in adipose tissue and the immune system that regulate genes in cell differentiation and in multiple metabolic processes [45]. Once activated the PPAR signaling pathway participates in the amplification of the inflammatory process. NOD-like receptors are cytoplasmic receptors that play a vital role in the innate immune system, recognizing molecular patterns associated with pathogens and damage. Activation of the NOD-like signaling pathway induces the innate immune response [46]. NF-κB is a protein complex that performs transcription factor functions, among them acting as a mediator of the inflammatory response regulation. Activation of NF-κB signaling pathway results in proinflammatory cytokine secretion [3]. Finally, the JAK-STAT signaling pathway that transduces intracellular signals is associated with inflammatory and autoimmune diseases [47]. *In vitro*, Li et al. showed artesunate could treat rosacea via the inhibition of the JAK/STAT signaling pathway [48]. These evidences strongly indicate lncRNAs play a role in the pathology of rosacea through the five pathways (ferroptosis, PPAR, NOD-like, NF-Κb, and JAK-STAT). In addition, lncRNAs and miRNAs may work together via JAK-STAT pathway.

Next, we showed that DE lncRNA NEAT1 was upregulated, while HOTAIR and ZNF667-AS1 were downregulated. NEAT1 is located on chromosome 11q13.1 and is expressed in various tissues and cell types. NEAT1 can dysregulate proinflammatory chemokines and cytokines in monocytes by mediating the TLR4-mediated inflammatory pathway in patients with SLE [40]. Further, NEAT1 is overexpressed in inflammatory bowel disease patients and participates in the inflammatory response by regulating the intestinal epithelial barrier and exosome-mediated polarization of macrophages [49]. Here, for the first time, it was identified a relationship between the NEAT1 and rosacea. Our data suggest that NEAT1 may cause damage in rosacea by regulating genes associated with immune dysregulation and inflammation, as indicated by NEAT1-regulated genes that are enriched in chemokine activity, which play crucial roles in inflammatory infiltration.

HOTAIR is located in the Homeobox C gene cluster on chromosome 12q13.13 and is important for suppressing the proliferation, invasion, migration, and tube formation of vein endothelial cells by downregulating the expression of vascular endothelial growth factor A [50]. Furthermore, HOTAIR is a critical participant in lipopolysaccharide-induced cytokine expression, immune and inflammatory response in macrophages [51]. This lncRNA also relieves inflammation by regulating the expression levels of inflammatory factors and inhibiting the activation of NF-κB pathway [52]. In our study, the downregulated expression of HOTAIR in all subtypes of rosacea indicates that HOTAIR may improve rosacea by alleviating inflammation and inhibiting the NF-kappa B pathway.

ZNF667-AS1, namely Zinc finger protein 667‐antisense RNA 1, is located on human chromosome 19q13.43 and is associated with cell proliferation, migration, and invasion [53]. In human normal cells, ZNF667-AS1 is highly expressed while reduced in immortalized cells and multiple tumor tissues [54]. Li et al. illustrated that ZNF667-AS1 could inhibit the inflammatory response and promote spinal cord injury recovery via suppressing the JAK-STAT pathway [55]. Although there is no research about the relationship between ZNF667-AS1 and rosacea, our data indicate that the downregulated ZNF667-AS1 may play an important role in rosacea. Whether ZNF667-AS1 prevents the development of rosacea via JAK-STAT pathway needs deep research.

## 5. Conclusions

In conclusion, this is the first study to identify the ceRNA network in rosacea. Pathways including ferroptosis, PPAR signaling pathway, NOD-like signaling pathway, NF-kappa B signaling pathway, and JAK-STAT signaling pathway can be considered important therapeutic targets for rosacea. The lncRNAs and mRNAs identified in our study may play key roles in rosacea development. Upregulated NEAT1 can be a possible biomarker for rosacea diagnosis as well as HOTAIR and ZNF667-AS1 can be a target for rosacea prevention and treatment. More in-depth researches are needed to better characterize the lncRNAs and mRNAs in rosacea.

## Data Availability

The data used to support the findings of this study are available from the corresponding author upon request.

## Supplementary Materials

Supplementary Materials See Figures S1–S3 in the Supplementary Material for comprehensive image analysis. Figure S1: (a) the enriched bubble diagrams of the GO analysis with differentially expressed genes of the lncRNAs in ETR group. The horizontal axis represents the proportion of those genes accounted for in all the GO annotated genes, the left side of the vertical axis represents the annotation terms, and the right side of the vertical axis represents biological process (BP) terms, cellular component (CC) terms, and molecular function (MF) terms. Bubble scale represents the number of genes in each GO term; depth of bubble color represents *p* value. (b) The three kinds of enriched GO biological process terms of differentially expressed mRNAs involved in the lncRNAs network in ETR group. Figure S2: (a) the enriched bubble diagrams of the GO analysis with differentially expressed genes of the lncRNAs in PhR group. The horizontal axis represents the proportion of those genes accounted for in all the GO annotated genes, the left side of the vertical axis represents the annotation terms, and the right side of the vertical axis represents biological process (BP) terms, cellular component (CC) terms, and molecular function (MF) terms. Bubble scale represents the number of genes in each GO term; depth of bubble color represents *p* value. (b) The three kinds of enriched GO biological process terms of differentially expressed mRNAs involved in the lncRNAs network in the PhR group. Figure S3: (a) the enriched bubble diagrams of the GO analysis with differentially expressed genes of the lncRNAs in the PPR group. The horizontal axis represents the proportion of those genes accounted for in all the GO annotated genes, the left side of the vertical axis represents the annotation terms, and the right side of the vertical axis represents biological process (BP) terms, cellular component (CC) terms, and molecular function (MF) terms. Bubble scale represents the number of genes in each GO term; depth of bubble color represents *p* value. (b) The three kinds of enriched GO biological process terms of differentially expressed mRNAs involved in the lncRNAs network in the PPR group.
