# Supplementary material for: Identification of Long Noncoding RNA Associated ceRNA Networks in Rosacea
Source: Biomed Res Int. 2020 Feb 24;2020:9705950. doi: 10.1155/2020/9705950 (PMC7060422; doi:10.1155/2020/9705950)
Supplement: Supplementary Materials — See Figures S1–S3 in the Supplementary Material for comprehensive image analysis. Figure S1: (a) the enriched bubble diagrams of the GO analysis with differentially expressed genes of the lncRNAs in ETR group. The horizontal axis represents the proportion of those genes accounted for in all the GO annotated genes, the left side of the vertical axis represents the annotation terms, and the right side of the vertical axis represents biological process (BP) terms, cellular component (CC) terms, and molecular function (MF) terms. Bubble scale represents the number of genes in each GO term; depth of bubble color represents p value. (b) The three kinds of enriched GO biological process terms of differentially expressed mRNAs involved in the lncRNAs network in ETR group. Figure S2: (a) the enriched bubble diagrams of the GO analysis with differentially expressed genes of the lncRNAs in PhR group. The horizontal axis represents the proportion of those genes accounted for in all the GO annotated genes, the left side of the vertical axis represents the annotation terms, and the right side of the vertical axis represents biological process (BP) terms, cellular component (CC) terms, and molecular function (MF) terms. Bubble scale represents the number of genes in each GO term; depth of bubble color represents p value. (b) The three kinds of enriched GO biological process terms of differentially expressed mRNAs involved in the lncRNAs network in the PhR group. Figure S3: (a) the enriched bubble diagrams of the GO analysis with differentially expressed genes of the lncRNAs in the PPR group. The horizontal axis represents the proportion of those genes accounted for in all the GO annotated genes, the left side of the vertical axis represents the annotation terms, and the right side of the vertical axis represents biological process (BP) terms, cellular component (CC) terms, and molecular function (MF) terms. Bubble scale represents the number of genes in each GO term; depth of bubb [file 9705950.f1.docx]

# Identification of long non-coding RNA associated ceRNA networks in rosacea

Running head: Identification of ceRNA networks in rosacea

Lian Wang,^1,†^ Ruifeng Lu,^2,†^ Yujia Wang,^1^ Xiaoyun Wang, Dan Hao,^1^ Xiang Wen,^1^ Yanmei Li,^1^ Minghui Zeng,^3,*^ Xian Jiang^1,*^

1 Department of Dermatology, West China Hospital, Sichuan University, Chengdu 610041, P. R. China

2 Department of Pediatrics, West China Second University Hospital, Sichuan University, Chengdu 610041, P. R. China

3 Department of Pharmacy, Qionglai Medical Center Hospital of Sichuan Province, Chengdu 611500, P. R. China

* Correspondence: jennyxianj@163.com; Tel.: +86-28-8164063

**Supplementary Materials**


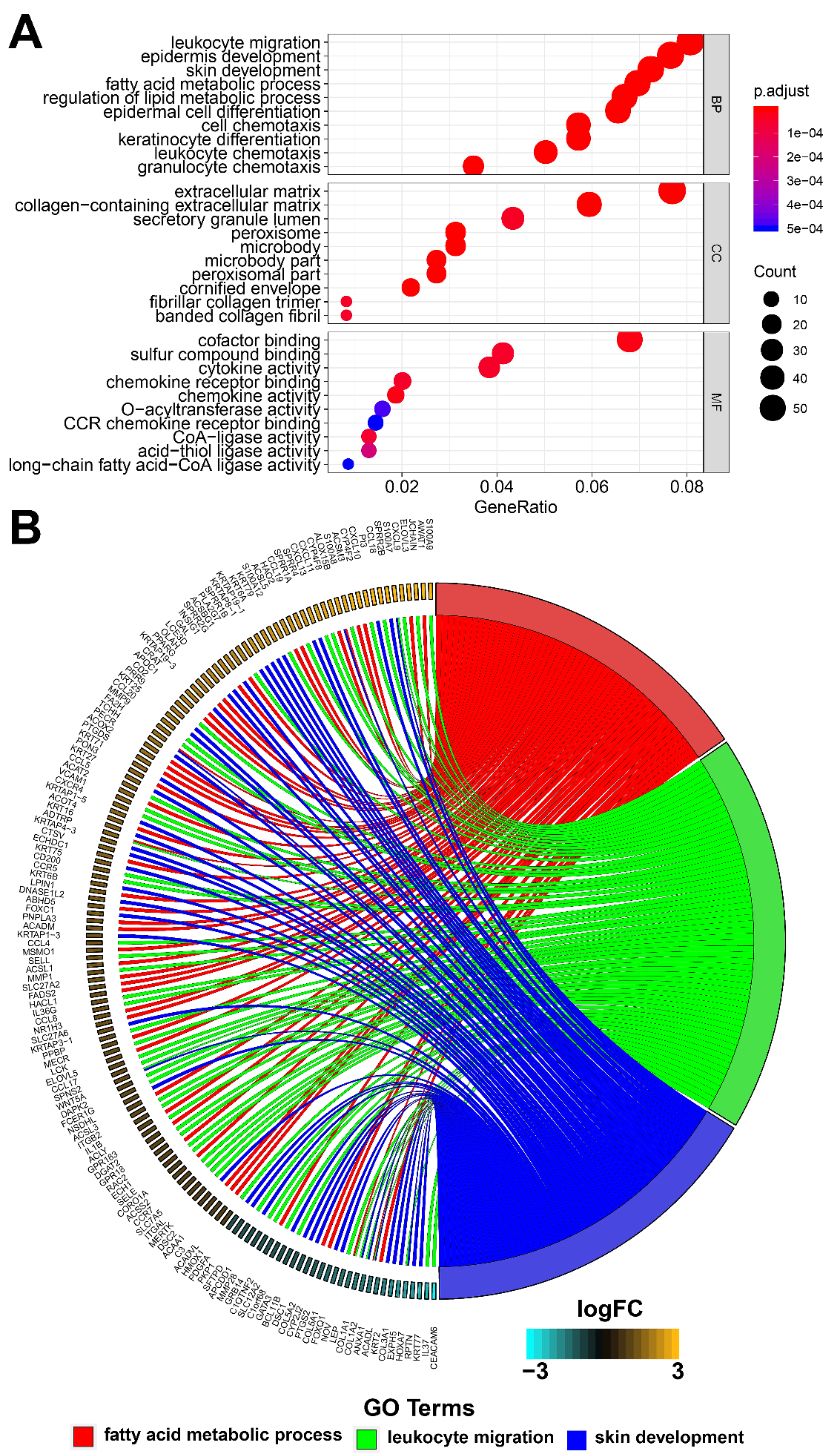


**Figure S1.** (a) The enriched bubble diagrams of the GO analysis with differentially expressed genes of the lncRNAs in ETR group. The horizontal axis represents the proportion of those genes accounted for in all the GO annotated genes, the left side of the vertical axis represents the annotation terms, and the right side of the vertical axis represents biological process (BP) terms, cellular component (CC) terms and molecular function (MF) terms. Bubble scale represents number of genes in each GO term; depth of bubble color represents p value. (b) The three kinds of enriched GO biological process terms of differentially expressed mRNAs involved in the lncRNAs network in ETR group.


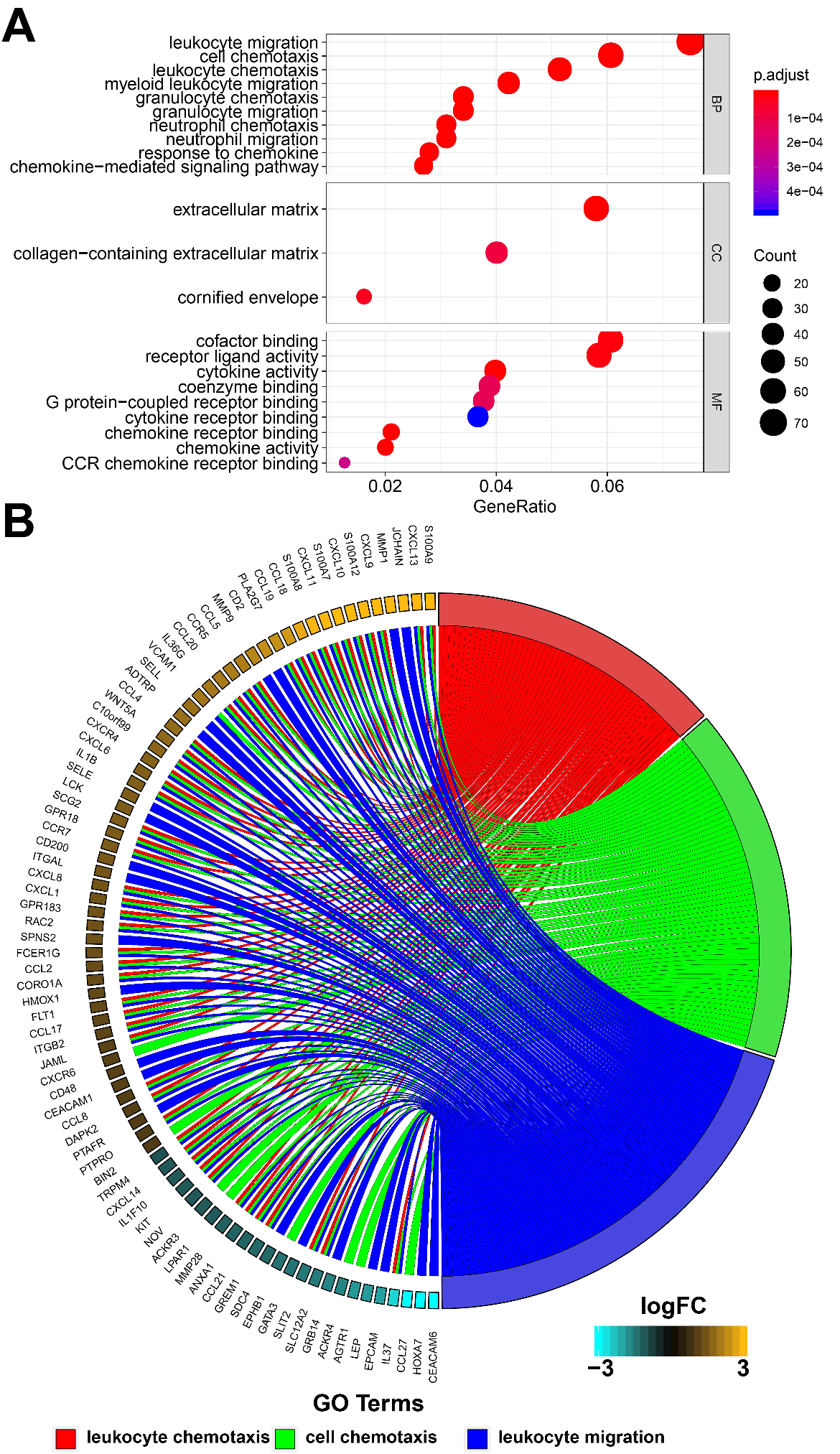


**Figure S2.** (a) The enriched bubble diagrams of the GO analysis with differentially expressed genes of the lncRNAs in PhR group. The horizontal axis represents the proportion of those genes accounted for in all the GO annotated genes, the left side of the vertical axis represents the annotation terms, and the right side of the vertical axis represents biological process (BP) terms, cellular component (CC) terms and molecular function (MF) terms. Bubble scale represents number of genes in each GO term; depth of bubble color represents p value. (b) The three kinds of enriched GO biological process terms of differentially expressed mRNAs involved in the lncRNAs network in PhR group.


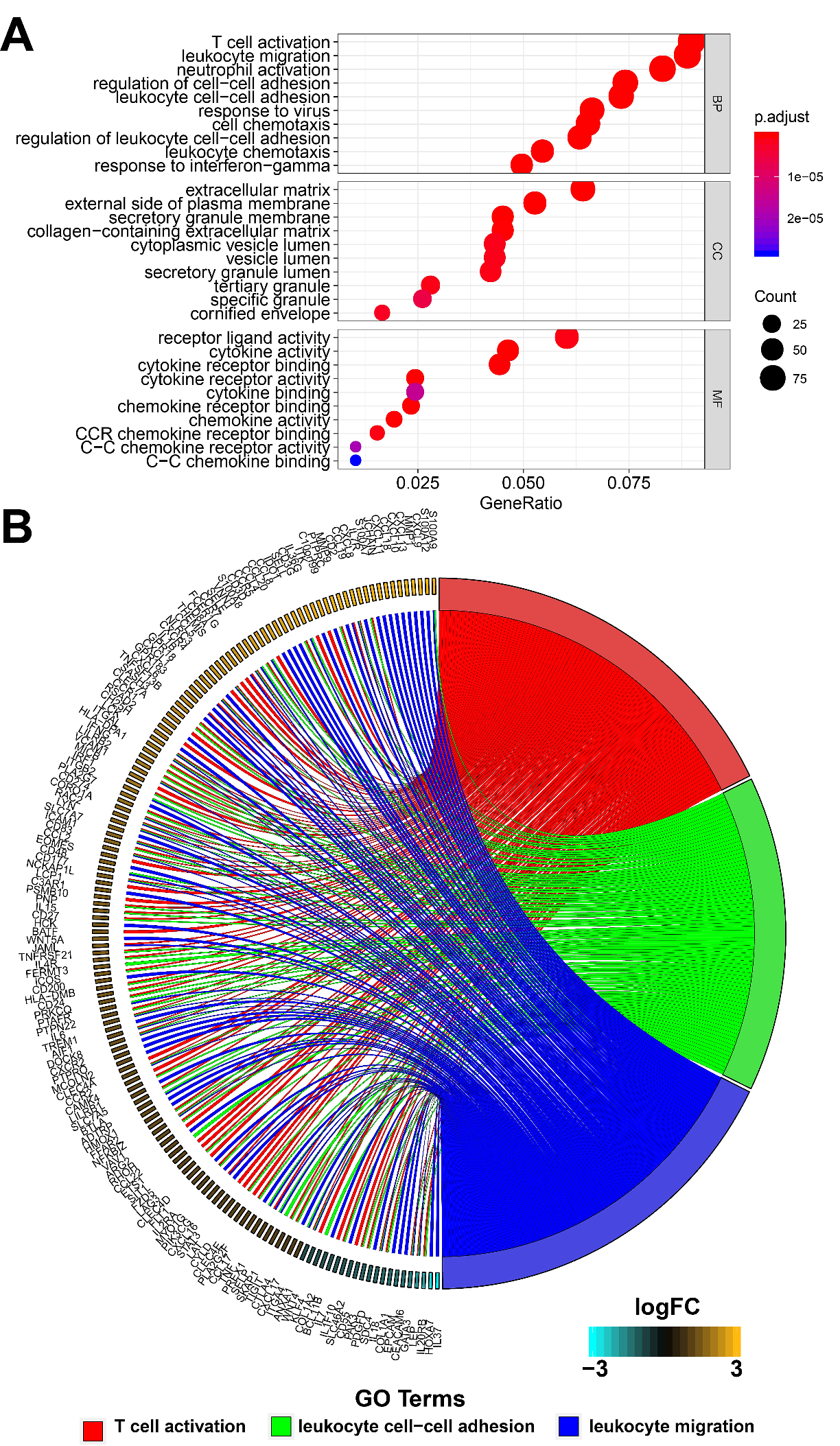


**Figure S3.** (a) The enriched bubble diagrams of the GO analysis with differentially expressed genes of the lncRNAs in PPR group. The horizontal axis represents the proportion of those genes accounted for in all the GO annotated genes, the left side of the vertical axis represents the annotation terms, and the right side of the vertical axis represents biological process (BP) terms, cellular component (CC) terms and molecular function (MF) terms. Bubble scale represents number of genes in each GO term; depth of bubble color represents p value. (b) The three kinds of enriched GO biological process terms of differentially expressed mRNAs involved in the lncRNAs network in PPR group.
